# Robust Simulations of Nanoscale Phase Change Memory: Dynamics and Retention

**DOI:** 10.3390/nano11112945

**Published:** 2021-11-03

**Authors:** Feilong Ding, Deqi Dong, Yihan Chen, Xinnan Lin, Lining Zhang

**Affiliations:** 1 School of Electronic and Computer Engineering, Peking University, Shenzhen 518055, China; 2001212758@stu.pku.edu.cn (F.D.); dqdong@sz.pku.edu.cn (D.D.); xnlin@pkusz.edu.cn (X.L.); 2 School of Humanities and Social Science, The Chinese University of Hong Kong, Shenzhen 518172, China; chenyihan@cuhk.edu.cn

**Keywords:** phase change memory, dynamic nucleation, fast SET programming, CMOS integrations, retention failure

## Abstract

A robust simulation framework was developed for nanoscale phase change memory (PCM) cells. Starting from the reaction rate theory, the dynamic nucleation was simulated to capture the evolution of the cluster population. To accommodate the non-uniform critical sizes of nuclei due to the non-isothermal conditions during PCM cell programming, an improved crystallization model was proposed that goes beyond the classical nucleation and growth model. With the above, the incubation period in which the cluster distributions reached their equilibrium was captured beyond the capability of simulations with a steady-state nucleation rate. The implications of the developed simulation method are discussed regarding PCM fast SET programming and retention. This work provides the possibility for further improvement of PCM and integration with CMOS technology.

## 1. Introduction

Phase change memory (PCM) is one of the emerging non-volatile memories with the most mature technology and the most industrialized prospects at present. It has the advantages of nanoscale cells, high reliability and endurance, high speed, and compatibility with CMOS technology [1]. In particular, integrations at the back-end-of-line of advanced CMOS technology brings exciting new features. The write speed of PCM can possibly be raised further to the sub-nanosecond level through material engineering [2,3] or innovative programming methods [4], comparable to other CMOS-based memories such as static random access memory (SRAM). One of the most widely studied phase change materials thus far is Ge_2_Sb_2_Te_5_(GST). One current concern for PCM is its reliability, e.g., the high-resistance amorphous state shifts to low-resistance states with unintended crystallization [5,6,7]. Nucleation at random sites and subsequent growth leading to the formation of percolation paths might be responsible for retention failure. Further theoretical and experimental studies are useful for maturity of the PCM technology, which enriches and enhances the CMOS technology in various applications such as storage class memory (SCM) and neuromorphic computing. 

From a theoretical perspective, nanoscale modeling and simulations play important roles in PCM technology advancements. Empirical formulas and Johnson–Mehl–Avrami (JMA) models [8] are widely used in compact models [9,10,11]. At the numerical simulation level, a classical nucleation and growth (NG) model proposed by Peng et al. [12] is commonly used to reflect the random nucleation and non-uniform distribution of phase states of materials. This model successfully takes the randomness of nucleation/growth and the distribution of phase states into consideration. Nucleation occurs in a small volume if the nucleus formation probability is significant. Growth occurs when there is a crystalline neighbor around the considered volume and the growth probability is high. Together with the NG model, simulations of PCM have been reported [13,14,15,16,17,18,19] by the Monte Carlo method based on geometry discretization from the finite element method. In [13], authors reported the simulation framework as well as the programming operations and the thermal boundary resistance effect. Another numerical simulation algorithm [14] was reported as a basis to propose a phase change module for multi-value storage capability. Further, the thermoelectric effects have been incorporated [15] in the coupling of thermal and electrical simulations. At the same time, simulations are also proved useful in the study of PCM variations and retention failures. By considering the stochastic nucleation, the intrinsic retention as well as its statistics, e.g., the cell-to-cell and cycle-to-cycle variability have been studied [16,17]. The phase field method [18,19] was later included in the simulation framework to address the crystallization from the perspective of total energy (bulk free-energy and interface energy) reduction. It has been shown that the retention time is improved for scaled PCM. However, one basic assumption behind the traditional simulations is a steady-state nucleation rate at the initial stage. The incubation period, i.e., the period in which the nuclei grow to their critical size, is not considered. On the other hand, a critical nuclei size is usually assumed upon nucleation for a discretized volume in the NG model. As such, the preferred volume size is equal to the critical size. Since the critical nuclei size is dependent on temperature [20], the NG model is more suitable for phase changes under a uniform temperature profile [20]. Alternative simulation frameworks are desired for nanoscale PCM.

In this work a simulation framework was developed for the evolution process of the nanoscale PCM embryo distribution from the perspective of dynamic nucleation. The classical nucleation growth model was improved to be suitable for the phase transition under non-uniform temperature conditions. Additionally, the relationship between the transient nucleation rate and the incubation period of the crystal nucleus was analyzed. The accelerating SET operation, as well as the device retention failure, was also discussed.

## 2. The Theory and Simulation Algorithm

**Figure 1:**
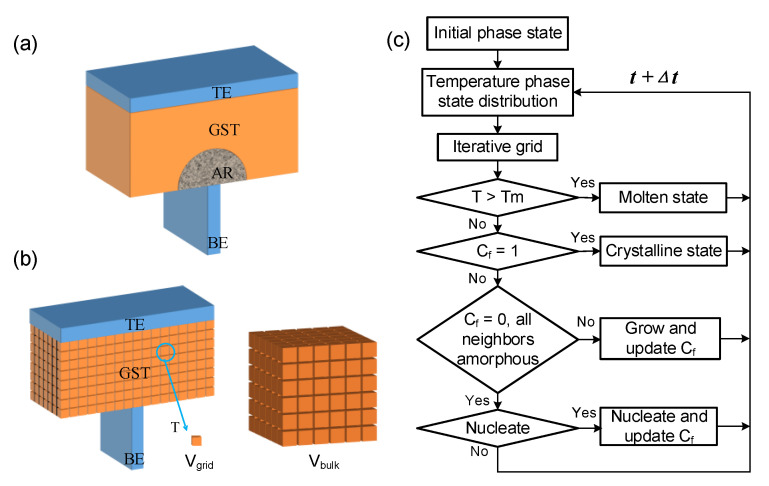
(**a**) PCM of the mushroom structure (TE and BE stand for top electrode and bottom electrode, respectively; AR represents active region); (**b**) schematic diagram of the structure of PCM cell and the calculation of random nucleation probability; (**c**) flow chart of the improved crystallization model simulation.

PCM of the classical mushroom type, as shown in Figure 1a, was used to demonstrate the simulation theory and algorithm.

### 2.1. Electrothermal Module

Joule heat is generated by applying electric pulses of different durations and amplitudes, which is used to realize the transformation of different phases of GST, which is the basic principle of PCM. The coupling of the current continuity equation and heat conduction equation is the key to building an electrothermal module:

(1) ∇J=-∇⋅(σ(∇V)=0

(2) $$\rho c_{p}\frac{dT}{dt}=\nabla \cdot (\kappa \nabla T)+\frac{J^{2}}{\sigma }$$

where *J* is the current density, *σ* is the electrical conductivity, *ρ* is the density of GST, *c_p_* is the heat capacity at constant pressure, *T* is the temperature, and *κ* is the thermal conductivity. To reproduce the correct thermal boundary, a large enough domain including the PCM cell and its surrounding dielectric is always simulated. The Dirichlet boundary condition is applied on the electrodes with its temperature of 293.15 K, and the Neumann boundary condition is applied on other surfaces. Thermal boundary resistance (TBR) at the interface between different materials, for example, the interface of GST/BE, is easily incorporated into the simulation. It facilitates a confinement of heating and hence the reduction of the reset current, which may be intentionally introduced in the PCM cell design [21,22]. Figure 1b briefly shows the discretization with cuboidal grids. The electrical conductivity of a mixed phase *σ_m_* is calculated with the Wiener upper bound model [23]:

(3) $$\sigma_{m}=\frac{\sigma_{c}\sigma_{a}}{C_{f}(\sigma_{a}-\sigma_{c})+\sigma_{c}}$$

where *σ_c_* and *σ_a_* are the electrical conductivity of the crystalline and amorphous states, respectively, and *C_f_* is the crystalline fraction of GST. The thermal conductivity of GST is divided into phonon thermal conduction and electronic thermal conduction. The electrical conductivity of the material is proportional to the thermal conductivity, which can be described by the Wiedemann Franz Law. The phonon thermal conductivity of the mixed state *κ_phm_* can be expressed as:

(4) $$\kappa_{phm}=C_{f}\kappa_{phc}+(1-C_{f})\kappa_{pha}$$

where *κ_phc_* and *κ_pha_* are the phonon thermal conductivity of the crystalline and amorphous states, respectively, and the thermal conductivity of the mixed phase *κ_m_* is

(5) $$\kappa_{m}=\kappa_{phm}+L_{0}T\sigma_{m}$$

where *L_0_* is the Lorenz number.

### 2.2. Dynamic Nucleation

The first step of crystallization requires the formation of new phase nuclei in the parent phase, similar to the classical nucleation and growth theory. The driving force for nucleation is that the volume free energy of the crystalline state is lower than the volume free energy of the amorphous state, and the resistance is due to the formation of a new surface by the crystal embryo, which will cause the surface energy *γ* to increase. The crystal embryo is approximately a sphere to reduce interface area; it will not become a stable crystal nucleus until the radius *r* of the crystal embryo exceeds the critical nucleation radius *r_c_*. After that, the nucleus can continue to grow. The expression for the number of molecules *n_c_* contained in the critical nucleation radius is

(6) $$n_{c}=\frac{32\pi \gamma^{3}}{3\Delta G_{V}{}^{3}V_{a}}$$

where *V_a_* is the average molecular volume of GST, and Δ*G_V_* is the free energy change per unit volume of the liquid-to-solid phase transition, which can be expressed as [24]

(7) $$\Delta G_{V}=\Delta H_{m}\frac{2T(T_{m}-T)}{T_{m}(T_{m}+T)}$$

where Δ*H_m_* is the latent heat, and *T_m_* is the melting temperature of the GST. A crystal embryo containing *n* molecules appears in the amorphous phase, and the total free energy difference Δ*G_n_* is

(8) $$\Delta G_{n}=4\pi r^{2}\gamma -nV_{a}\Delta G_{V}$$

where *r* is radius of the embryo and can be expressed as

(9) $$r={\left(\frac{3nV_{a}}{4\pi }\right)}^{1/3}$$

The crystal embryo in the amorphous phase may increase its size with more molecules, or it may decrease its size with less molecules due to dissolution. The expansion and dissolution processes occur in parallel and are described by the following two expressions [14]:

(10) $$A_{n-1}+A_{1}\overset{P_{n-1}^{+}}{\underset{P_{n}^{-}}{⇄}}A_{n}$$

(11) $$A_{n}+A_{1}\overset{P_{n}^{+}}{\underset{P_{n+1}^{-}}{⇄}}A_{n+1}$$

where *A*_1_, *A*_*n*−1_, *A_n_*, and *A*_*n*+1_ are embryos containing 1, *n* − 1, *n*, and *n* + 1 molecules, respectively, and $P_{n}^{+}$ is the probability that the embryo *A_n_* will increase by one molecule. $P_{n}^{-}$ is the probability that the embryo *A_n_* will decrease by one molecule. The transient expression *N_n,t_* of the number of embryos containing n molecules in the system at time *t* is, then [25],

(12) $$N_{n,t}=N_{n,t-\Delta t}+\Delta t\cdot (P_{n-1}^{+}N_{n-1,t-\Delta t}-P_{n}^{-}N_{n,t-\Delta t}-P_{n}^{+}N_{n,t-\Delta t}+P_{n+1}^{-}N_{n+1,t-\Delta t})$$

(13) $$P_{n}^{+}=\frac{O_{n}D}{\lambda^{2}}\exp(-\frac{△g_{n}}{2k_{B}T})$$

(14) $$P_{n}^{-}=\frac{O_{n}D}{\lambda^{2}}\exp(\frac{△g_{n}}{2k_{B}T})$$

where *O_n_* is the number of molecules on the surface of the embryo *A_n_*, which is approximately *O_n_* ≈ 4*n*^2/3^; *D* is the diffusion coefficient; *λ* is the atomic jump distance; and Δ*g_n_* is the amount of energy change in the system after the embryo *A_n_* gains or loses one molecule. The nucleation rate at any time can be obtained by solving the grain flux:

(15) $$I_{n,t}=P_{n}^{+}N_{n,t}-P_{n+1}^{-}N_{n+1,t}$$

With the passage of annealing time, the number of embryos will gradually increase, and the size will continue to increase, and the nucleation rate will continue to rise, but at the same time, more embryos will dissolve, and the number of embryos will develop towards a steady state, while the nucleation rate reaches the steady-state value $I_{nc}^{s}$.

After nucleation, under the driving force of crystallization, the crystal nucleus will grow up. Since the mobility of molecules is affected by temperature, its growth rate is affected by temperature. Kelton et al. developed the GST grain growth rate *v_g_* suitable for a wide temperature range [26]:

(16) $$v_{g}(T)={\left(\frac{3V_{a}}{4\pi }\right)}^{1/3}\frac{D}{3n_{c}{}^{2/3}}\left\{1-\exp\left[\frac{\Delta G_{V}}{k_{B}T}\left(\frac{r_{c}}{r}-1\right)\right]\right\}$$

### 2.3. The Algorithm and Improved Crystallization Model

On the basis of the above model framework, a new algorithm was developed, as shown in Figure 1b. According to the principle of equal distribution of the sample and the population, a dual grid technique was proposed to solve the dynamic nucleation based on the Monte Carlo method. Unlike the classical nucleation growth model, which regards the grid as the element of the crystal nucleus, this model only regards the grid as the carrier of the crystal nucleus, and the change of the grid crystalline fraction is obtained by solving the ratio of the crystallized volume change to the grid volume. The steps are as follows: First, the GST region is divided into many small grids with a volume of *V_grid_* (*V_grid_ =* $d_{grid}^{3}$). It is assumed that the temperature in the grid is uniformly distributed and considers the grid as a part of a large volume of *V_bulk_* whose temperature is uniform. *n_grid_* (*n_grid_ = V_bulk_/V_grid_*) is the number of samples in the population, and the number of nuclei in the population in a time step Δ*t* can be calculated as $N_{bulk}^{c}$ ($N_{bulk}^{c}$ *=*$I_{nc}^{s}$*∙*Δ*t∙V_bulk_*); then, the probability *P_n_* of crystal nuclei appearing in the small grid is

(17) $$P_{n}=\frac{N_{bulk}^{c}}{n_{grid}}=\frac{I_{n_{c}}^{s}\cdot \Delta t\cdot V_{bulk}}{V_{bulk}/V_{grid}}=I_{n_{c}}^{s}\cdot \Delta t\cdot V_{grid}$$

The probability of the appearance of a crystal nucleus is combined with the Monte Carlo algorithm to simulate the nucleation process. The flow chart is shown in Figure 1c. If a crystal nucleus appears in the grid, the size of the crystal nucleus is recorded as the critical nucleation. The crystalline fraction of the updated mesh due to nucleation is

(18) $$C_{f,t}=\frac{n_{c}V_{a}}{V_{grid}}$$

If the volume of the crystal nucleus is smaller than the volume of the grid, record the size of the crystal nucleus; if the volume of the crystal nucleus exceeds the volume of the grid, the surrounding grid will evenly divide the grid and the crystalline fraction of the grid needs to be updated. The crystalline fraction of the updated mesh due to growth is

(19) $$C_{f,t}=C_{f,t-\Delta t}+\frac{4\pi {\left(r_{t-\Delta t}+v_{g}\Delta t\right)}^{3}}{V_{grid}}+\frac{N_{c}\cdot v_{g}\cdot \Delta t\cdot d_{grid}^{2}}{V_{grid}}$$

where *r*_*t*−Δ*t*_ is the radius of the crystal nucleus at time *t* − Δ*t*, and *N_c_* is the number of nearest surrounding grids with *C_f_* = 1.

### 2.4. Simulation Robustness

**Figure 2:**
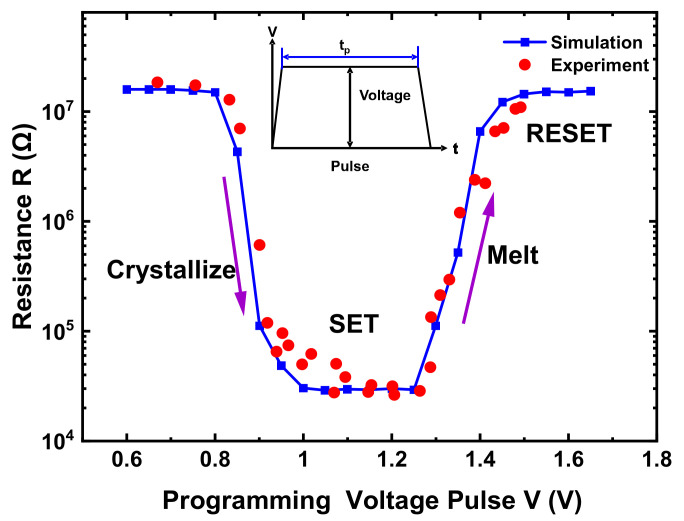
Simulation and experiment results [15] of PCM cell resistances vs. the programming voltage. Voltage pulses with fixed width *t_p_* = 170 ns and increasing amplitudes are applied.

Figure 2 shows PCM R–V characteristics with an amorphous initial state. Programming voltage pulses with different amplitudes and fixed width *t_p_* = 170 ns were applied to the PCM cell, in accordance with [15]. The resistance value was obtained by a reading following each programming pulse. With the increasing of voltage amplitudes, the PCM cell went through the SET to low resistance and then the RESET to high resistance. The simulation results agreed reasonably with the experimental data. In particular, the partial RESET of varying degrees was well reproduced.

**Figure 3:**
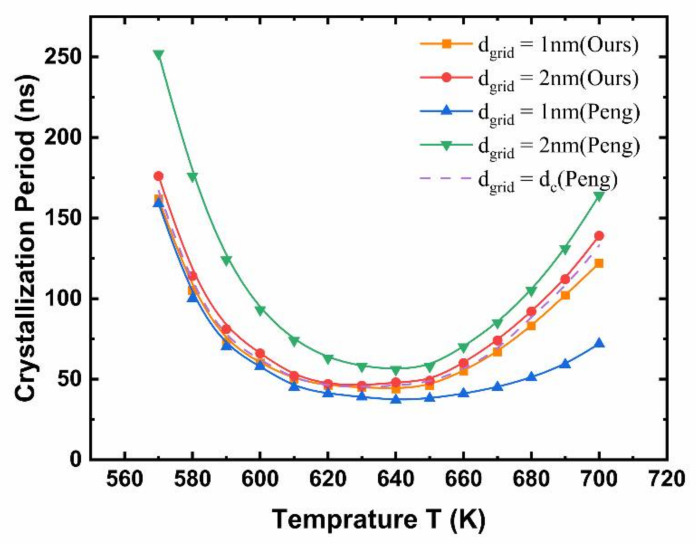
The crystallization time curve of the improved crystallization model and the classical nucleation growth model at different annealing temperatures and grid sizes.

Figure 3 shows the simulated crystallization period as a function of the annealing temperature from the improved crystallization model and the classical nucleation growth model. The results are indeed the average values of multiple measurements. Different grid sizes were set up for the purpose of robustness verification. The purple dotted line represented results of the classical nucleation growth model under the critical nucleation size (which changes according to the temperature). Two other grid sizes, 1 nm and 2 nm, were also used for comparison. With the classical NG model, it could be seen that at the same temperature, the selection of the grid size had a greater impact on the crystallization period, because it is unreasonable to set a uniform grid size for different temperatures corresponding to different critical nucleation sizes. In contrast, the improved crystallization model gave more stable results without significant impacts from grid size, and the results were also close to those of the NG model with a temperature-dependent grid size.

### 2.5. The Incubation Period

**Figure 4:**
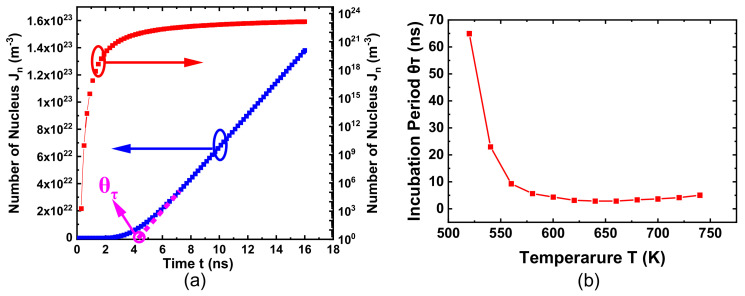
(**a**) The total number of nuclei as a function of time at 700 K, assuming that there is no embryo in the initial state (including log coordinates and linear coordinates). The slope in the linear coordinate is the steady-state nucleation rate, and the linear extrapolation gives the incubation period from the intersection point. (**b**) Incubation period at different temperatures, assuming that there is no embryo in the initial state.

Assuming that there are no embryos in the initial state, the function relationship between the number of nucleus *J_n_* and time *t* is shown in Figure 4a. It can be seen that as time goes by, the number of crystal nuclei gradually increases from zero. It gradually stabilizes, indicating that the steady-state nucleus rate is reached. The *θ_τ_* in Figure 4a is the nucleus incubation period, and Figure 4b shows the nucleus incubation period at different temperatures, assuming that there is no embryo in the initial state. With the increase in temperature, the incubation period first decreases because of the increase in the mobility of molecules and the probability of their collision. The incubation period then increases slightly, most probably because of the decrease in the degree of subcooling and the increase in the critical nucleation radius, which requires a longer time to reach the critical nucleation molecule number.

**Figure 5:**
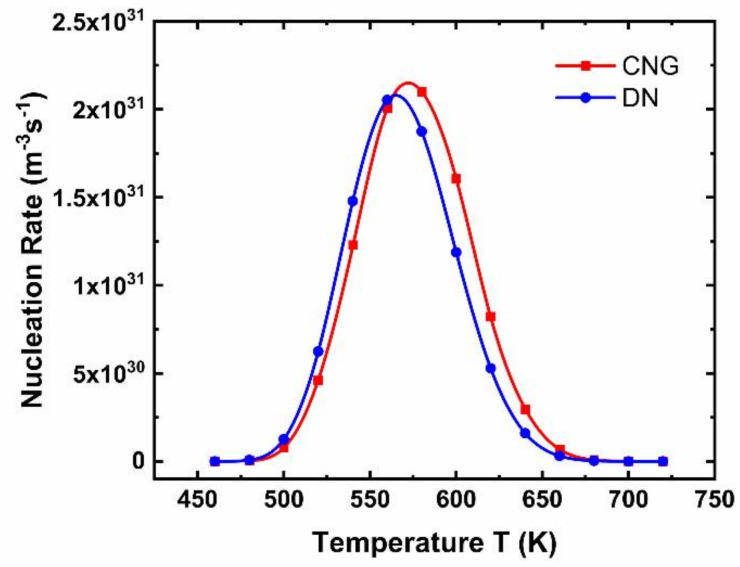
Comparison of the steady-state rate of dynamic nucleation (DN) and that of classical nucleation growth model (CNG) under different temperatures.

Figure 5 shows that dynamic nucleation in the steady-state and the traditional steady-state nucleation rate are in good agreement with a slight deviation at the peak value, and the optimal nucleation temperature corresponding to the peak slightly changes. Nucleation rates in Figure 5 correspond to the slope of linear coordinates in Figure 4a.

## 3. Implication on PCM Cell Operations

Based on the above simulation framework, PCM operations were simulated in this section. The default geometry parameters used are summarized in Table 1.

**Figure 6:**
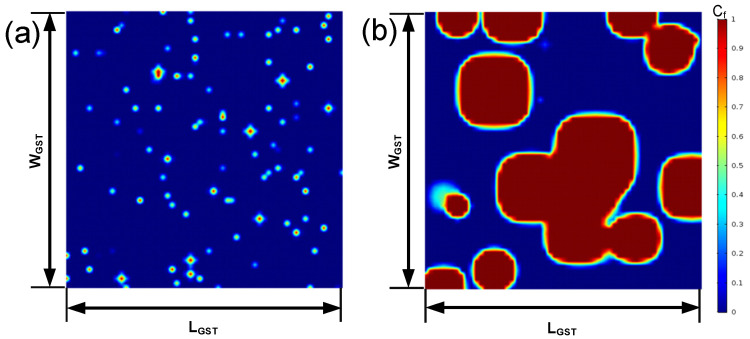
Crystallization after annealing at different temperatures, namely (**a**) 580 K and (**b**) 660 K, for 30 ns, assuming that there is no embryo in the initial state.

Figure 6 shows the crystallization conditions after annealing at 580 K and 660 K for 30 ns, assuming that there is no embryo in the initial state. In Figure 6a, a large number of small grains were formed, and in Figure 6b, a smaller number of grains was observed with larger sizes. The incubation periods were 5.6 ns and 2.9 ns under 580 K and 600 K, respectively. In the simulation period of 30 ns, the total number of nucleus depended on the slope in Figure 4a, which was higher under 580 K. The growth rate as given by Equation (6) was enhanced under 660 K for a large crystalline fraction.

**Figure 7:**
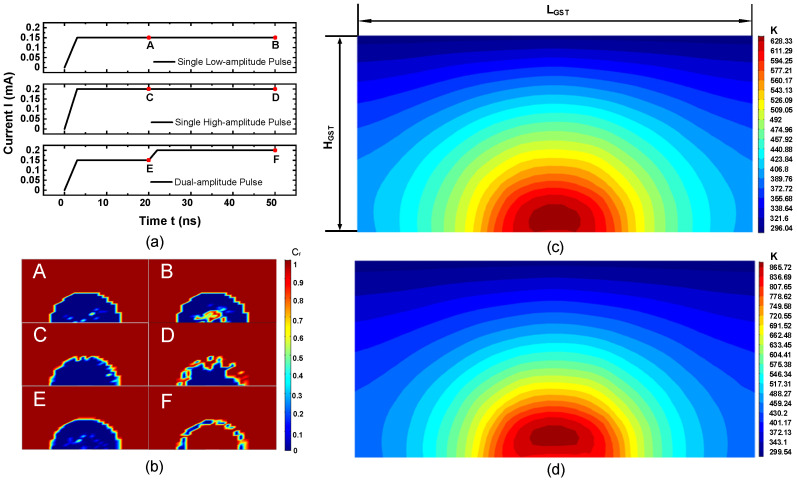
(**a**) Three SET operation pulse schemes; (**b**) the phase distribution corresponding to the corresponding point A–F (A, B correspond to a single low-amplitude pulse; C, D correspond to a single high-amplitude pulse; E, F correspond to a dual-amplitude pulse). (**c**) The instantaneous temperature profile corresponds to A of low amplitude pulse, and (**d**) the temperature profile corresponds to D of the high-amplitude case.

Figure 7 shows the application of three different SET pulse schemes and the phase distribution corresponding to points A–F. Applying a low-amplitude pulse will rapidly nucleate the inside of the active area, but due to the low temperature inside the active area, the growth rate of the formed nuclei slows down and the active area cannot be crystallized quickly. The case is shown as the top phase maps A and B in Figure 7b. Applying a higher amplitude pulse, although the growth rate of the crystal nucleus is faster at this temperature, the active area cannot be crystallized quickly from inside due to a lower nucleation rate. The case is shown as the middle phase maps C and D in Figure 7b. Applying a dual-amplitude pulse, a lower-amplitude pulse promotes the nucleation process, and then a higher-amplitude conventional SET pulse promotes the rapid growth of the crystal nucleus and crystallizes almost the entire active region. The case is shown as the bottom phase maps E and F in Figure 7b. The instantaneous temperature profile for A of low-amplitude pulse case and D of high-amplitude pulse case are shown in Figure 7c,d.

**Figure 8:**
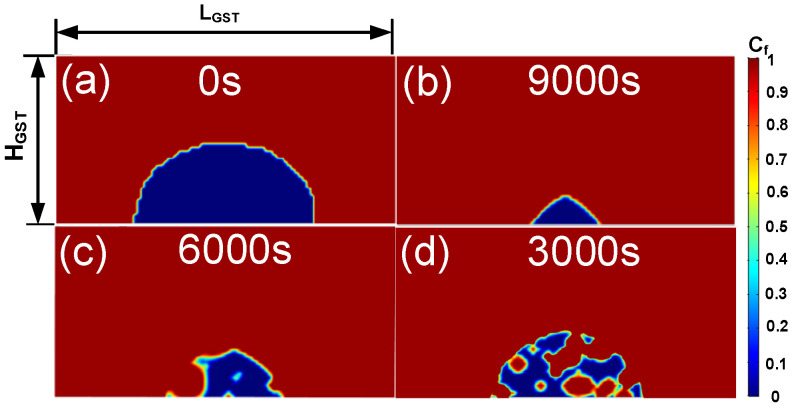
After RESET, the cell was annealed at a constant temperature of 450 K assuming different amounts of crystal embryo. (**a**) Phase distribution at 0 s; (**b**) phase distribution after annealing for 9000 s, without considering the initial state of the crystal embryo; (**c**) phase distribution after annealing for 6000 s, with considering the crystal embryos produced during quenching; (**d**) phase distribution after annealing for 3000 s, with considering more crystal embryos.

Figure 8 plots the annealing of a PCM cell, assuming different amounts of crystal embryos for the initial states. In the simulation, the cell was annealed under a constant temperature of 450 K. Figure 8a shows the phase distribution at the initial moment with a defined active region. Figure 8b is the phase distribution after annealing for 9000 s, without considering the embryos generated in the previous quenching process. The retention process was mainly due to the growth from the outer to inward. This was because when the embryos produced during cooling were not considered, the embryos’ incubation periods were longer, and the transient nucleation rate was lower. Figure 8c is the phase distribution after annealing for 6000 s. The failure process of the device was due to both the growth of the outer edge crystal and the nucleation of the amorphous region. The retention failure is accelerated compared with Figure 8b. Figure 8d is the phase distribution after annealing for 3000 s when an even larger number of embryos was considered. The nucleation of the amorphous region was dominant, leading to a percolation path with early failure. In other words, the initial nucleation rate was close to a steady-state rate, and the device would fail prematurely due to the large number of nuclei in the active region.

The simulation implementation and efficiency were summarized briefly. MATLAB and COMSOL were used jointly to implement the simulation flow of Figure 1c. While MATLAB was used for meshing and dynamic nucleation calculation with the in-house code, COMSOL was used for the solutions of electrothermal formulations. On a workbench with a CPU (intel(R) Core (TM) i7-4790 @ 3.60 GHz), it took about 7 h to obtain the data point shown in Figure 2, and about 0.5 h to finish the annealing under a constant temperature, shown in Figure 6.

## 4. Conclusions

A simulation framework was developed to study the operation of PCM cell operations. The dynamic nucleation process was implemented with the reaction rate theory. An improved crystallization model was also developed with robustness regarding the choice of grid size. Together with the electrothermal module, the PCM simulation framework was used to guide the PCM operations. The incubation period was captured with the developed method, including descriptions of the embryo distributions. A dual-amplitude pulse scheme was then analyzed to accelerate the SET process, i.e., using a lower temperature to induce enough nucleus, and then using a higher temperature to enhance the growth rate. The different failure mechanisms of the device caused by different embryo distributions were analyzed, which may explain the early retention failure driven by nucleation.

## Figures and Tables

**Table 1:** Default geometry parameters of simulated PCM cells.

Symbol	Description	Value	Unit
***W_GST_***	width of GST region	190	nm
***L_GST_***	length of GST region	190	nm
***H_GST_***	height of GST region	90	nm

## Data Availability

Not applicable.
